# Quantitative perfusion and water transport time model from multi *b*-value diffusion magnetic resonance imaging validated against neutron capture microspheres

**DOI:** 10.1117/1.JMI.10.6.063501

**Published:** 2023-12-08

**Authors:** Mira Liu, Niloufar Saadat, Yong Jeong, Steven Roth, Marek Niekrasz, Mihai Giurcanu, Timothy Carroll, Gregory Christoforidis

**Affiliations:** a University of Chicago, Committee on Medical Physics, Department of Radiology, Chicago, Illinois, United States; b University of Illinois, Department of Anesthesiology, Chicago, Illinois, United States; c University of Chicago, Department of Statistics, Chicago, Illinois, United States

**Keywords:** magnetic resonance imaging, perfusion, diffusion, stroke, cerebrospinal fluid

## Abstract

**Purpose:**

Quantification of perfusion in ml/100 g/min, rather than comparing relative values side-to-side, is critical at the clinical and research levels for large longitudinal and multi-center trials. Intravoxel incoherent motion (IVIM) is a non-contrast magnetic resonance imaging diffusion-based scan that uses a multitude of b-values to measure various speeds of molecular perfusion and diffusion, sidestepping inaccuracy of arterial input functions or bolus kinetics. Questions remain as to the original of the signal and whether IVIM returns quantitative and accurate perfusion in a pathology setting. This study tests a novel method of IVIM perfusion quantification compared with neutron capture microspheres.

**Approach:**

We derive an expression for the quantification of capillary blood flow in ml/100 g/min by solving the three-dimensional Gaussian probability distribution and defining water transport time (WTT) as when 50% of the original water remains in the tissue of interest. Calculations were verified in a six-subject pre-clinical canine model of normocapnia, ${\mathrm{CO}}_{2}$ induced hypercapnia, and middle cerebral artery occlusion (ischemic stroke) and compared with quantitative microsphere perfusion.

**Results:**

Linear regression analysis of IVIM and microsphere perfusion showed agreement (slope = 0.55, intercept = 52.5, $R^{2}=0.64$) with a Bland–Altman mean difference of −11.8 [−78,54]  ml/100  g/min. Linear regression between dynamic susceptibility contrast mean transit time and IVIM WTT asymmetry in infarcted tissue was excellent (slope=0.59, intercept = 0.3, $R^{2}=0.93$). Strong linear agreement was found between IVIM and reference standard infarct volume (slope = 1.01, $R^{2}=0.79$). The simulation of cerebrospinal fluid (CSF) suppression via inversion recovery returned a blood signal reduced by 82% from combined T1 and T2 effects.

**Conclusions:**

The accuracy and sensitivity of IVIM provides evidence that observed signal changes reflect cytotoxic edema and tissue perfusion and can be quantified with WTT. Partial volume contamination of CSF may be better removed during post-processing rather than with inversion recovery.

## 1 Introduction

Intravoxel incoherent motion (IVIM) is a post-processing algorithm for diffusion magnetic resonance imaging (MRI) scans introduced by Le Bihan et al.;^1^ it uses multiple b-values to simultaneously assess tissue water diffusion and tissue perfusion. Prolonged scan times, ease of use, and direct quantification of perfusion afforded by contrast-enhanced scans largely supplanted IVIM as a clinical option. However, because newer, faster scanner hardware and software have substantially reduced scan and image processing times, many groups^2^^–^^6^ are beginning to revisit IVIM perfusion-diffusion images as a tool for evaluating neurovascular disease. If IVIM can be established as a reliable, quantitative scan protocol that requires under 5 min of scan time and no contrast, quantitative perfusion can be added to any neurovascular scan protocol. This could provide cross-sectional and longitudinal tissue perfusion maps to allow physicians to ascertain changes in perfusion that are associated with chronic disease progression, such as intracranial atherosclerotic disease, carotid stenosis, and moyamoya disease, as well as treatment response.^7^^–^^9^ Furthermore, if cerebral blood flow can be quantified in ml/100 g/min (qCBF) without organ-dependent capillary segment length and dimension assumptions, IVIM has the potential to allow multi-site cross-sectional comparison in addition to within-patient longitudinal comparison in organs other than the brain.^10^^–^^12^

The IVIM sequence and post-processing presented here presents a clinically feasible scan time of 5-min to collect images for the calculation of parametric perfusion, diffusion, and water transport time (WTT) MR images. We hypothesize that IVIM perfusion values from multi-b-value diffusion with a standard IVIM bi-exponential can be quantified (ml/100 g/min) using WTT, agree with neutron capture microspheres in a setting of altered hemodynamics, and demonstrate similar sensitivity to physiologic changes. As the central volume principle of blood flow being equal to blood volume/transit time is predicated on the passage of bolus, by comparison of an IVIM model that assumes instantaneous bolus mimicking the central volume principle through isotropic Gaussian diffusion, we hypothesize that IVIM WTT should correlate with dynamic susceptibility contrast (DSC) mean transit time (MTT). Agreement would further answer questions of if observed “perfusion” changes are reflecting the flow of the perivascular cerebrospinal fluid (CSF) or the movement of blood in the capillary bed.^13^ Further, using the same IVIM acquisition, we hypothesize that IVIM diffusion positive infarct volumes will be accurate compared to MRI mean diffusivity (MD) infarct volumes as a reference standard. The purpose of this study is thus to assess the accuracy and sensitivity of a novel quantification of 5-min simultaneous IVIM perfusion and diffusion across three physiologic states.

## 2 Materials and Methods

### 2.1 IVIM Quantification via Water Transport Time

Perfusion and diffusion values are calculated from the multiple b-value IVIM scan using software developed in-house with a two-step fitting algorithm built using previous literature.^1^^,^^10^^,^^11^ In our implementation, the diffusion regime ($b>222\;s/{\mathrm{mm}}^{2}$) is fit to the mono-exponential $(1-f)e^{-Db}$, and the perfusion regime is fit to the remainder $fe^{-D^{*}b}$, resulting in the following standard bi-exponential^1^^,^^10^^,^^11^^,^^14^ on a voxel-by-voxel basis:

$$\frac{S(b)}{S(0)}=fe^{-D^{*}b}+(1-f)e^{-Db},$$ (1)

where $D^{*}$ is the pseudo-diffusion coefficient, f is the blood volume fraction (CBV), and D is the diffusion coefficient.^6^

With the bi-exponential fit returning an approximation of a pseudo-diffusion coefficient $D^{*}$ representing the fast component of water motion, the movement of perfusing water can be modeled as a one-dimensional Gaussian. WTT is then defined as the time for 50% of the original perfusing water to have left a voxel. With three directions taken, this can be written in spherical coordinates for three-dimensional (3D) pseudo-diffusion, set equal to 0.5 and solved for WTT as

$$\frac{4\pi }{{\left(\sqrt{4\pi D^{*}\mathrm{WTT}}\right)}^{3}}\int_{0}^{.5}e^{-\frac{r^{2}}{4D^{*}(\mathrm{WTT})}}r^{2}dr=0.5.$$ (2)

Quantitative CBF can be calculated by substituting this definition of WTT into the central volume principle, where WTT is solved from Eq. (2) as a function of $D^{*}[{\mathrm{mm}}^{2}/s]$, as shown in the Appendix. Fast-water fraction (f) per WTT (t) is proportional to the cerebral blood flow in ml/100 g/min with assumed values of ρ=1.04  g/mL and water content fraction $f_{w}=0.79$, which is converted to ml/100 g/min as

$$q\mathrm{CBF}[\frac{\frac{\mathrm{ml}}{100\;g}}{\min}]=\frac{\mathrm{CBV}}{\mathrm{WTT}}=\frac{f\times f_{w}}{\rho }\frac{2D^{*}}{{\left(.32\right)}^{2}}\approx fD^{*}\times 93,000.$$ (3)

Using Eq. (3) on IVIM bi-exponential parameters from Eq. (1) returns IVIM maps of qCBF in ml/100 g/min for comparison to reference standard values.

### 2.2 Comparison with Capillary Geometry

Quantitative perfusion values derived from Eq. (3) were compared directly to those derived based on capillary geometry.^10^ Quantification by capillary geometry requires an assumed water fraction of 0.65, density of 1.047  g/mL, total capillary length of 2 mm, and average segment length = 0.11 mm. We performed a regression analysis to ascertain agreement with reference standard neutron capture microspheres in the same IVIM images

### 2.3 Pre-Clinical Model

All experiments were conducted using a pre-clinical canine model of both controlled hypercapnia and MCAO.^15^^,^^16^ The 2-day experimental protocol was approved by the University of Chicago Institutional Animal Care and Use Committee and reported in compliance with ARRIVE guidelines. The University of Chicago is an AAALAC International accredited institution adhering to the following guidelines, regulations, and policies: (a) Guide for the Care and Use of Laboratory Animals (National Research Council), (b) USDA Animal Welfare Act and Animal Welfare Regulations, and (c) Public Health Service Policy on Humane Care and Use of Laboratory Animals. The canine model has several advantages compared to rodents as a model for assessment of infarct progression using perfusion and diffusion imaging studies. Canines have a gyrencephalic neocortex with similar ratio of white to gray matter^17^^–^^19^ as well as comparable pial arteriolar network organization,^20^ critical in modeling collateral arterial blood supply and predicting infarct evolution,^21^ to humans. In addition, the canine model provides tissue volumes necessary to evaluate both core and penumbra during ischemia using microsphere deposition. The neurovascular structure of canines also allows a range of endovascular devices and interventional radiology techniques allowing minimal invasion with real-time visualization of occlusion, preventing imaging artifacts and traumatic cerebrovascular reaction from open surgical occlusion. Finally, the middle cerebral artery occlusion proposed is a relatively inexpensive alternative to nonhuman primate models of acute ischemia.

### 2.4 Experimental Protocol

In each animal, two experiments were performed on consecutive days to validate our algorithm in a setting of cerebrovascular response to hypercapnia and MCAO and the effect of flow augmentation on collateral arterial networks after MCAO.^15^^,^^21^ We present an analysis of these experiments in which neutron capture microspheres and IVIM were acquired during quiescent physiologic conditions. On the first day, canines were imaged at baseline (i.e., normocapnia) (target ${\mathrm{PaCO}}_{2}=30$ to 35 mmHg) and hypercapnia (5% to 7% carbogen respiration, target ${\mathrm{PaCO}}_{2}$ 60 to 70 mmHg) induced by carbogen gas inhalation (5% to 7% ${\mathrm{CO}}_{2}$ 95% $O_{2}$). End tidal ${\mathrm{CO}}_{2}$ was monitored to assess minute to minute variations in exhaled ${\mathrm{CO}}_{2}$ levels, and true arterial ${\mathrm{paCO}}_{2}$ was measured through direct arterial blood sampled from the descending aorta at the time of perfusion measurements.

On the second day, canines underwent permanent endovascular middle cerebral artery occlusion under fluoroscopic guidance. Occlusion was then verified via selective ipsilateral and contralateral internal carotid and vertebral arteriography.^15^ Subjects were transported to the MRI suite for imaging studies within 60 min of occlusion. We evaluated experimental flow augmentation during MCAO via simultaneous administration of a vasopressor and vasodilator intended to augment CBF;^22^ therefore, highly dynamic and abnormally high perfusion throughout the brain post-occlusion is expected by experimental design. Throughout the experiments, physiologic parameters were maintained within normal range (excluding ${\mathrm{PaCO}}_{2}$ for experiments involving intentional hypercapnia and blood pressure for experiments involving flow augmentation).

IVIM and microspheres included in the study were taken <30  min apart on day two post-occlusion to mitigate perfusion fluctuation. Furthermore, physiologic parameter variation (heart rate, blood pressure, ${\mathrm{ETCO}}_{2}$) between microsphere and IVIM imaging was monitored. Large variance in physiologic parameters within these timing windows is noted, and subjects were removed from analysis [Fig. 3(b)] due to the confounding effect.

### 2.5 Microsphere Acquisition

**Fig. 1:**
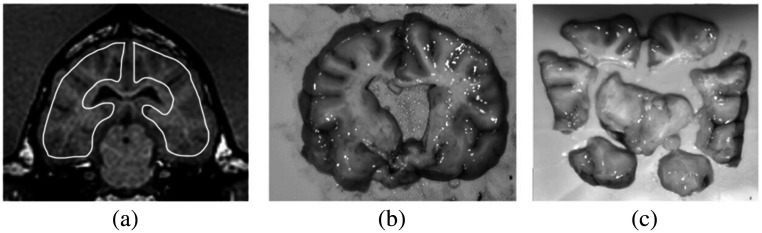
(a) Representative images for hemispheric ROIs on one of three slices drawn on IVIM and on DSC on an anatomic T1 weighted MRI. Edges are avoided to avoid subarachnoid CSF and DSC susceptibility artifacts. The brain slice (b) is cut into eight regions (c) for microsphere analysis and averaged for hemispheric qCBF.

Stable-isotope microsphere CBF in ml/100 g/min was acquired using neutron activation.^15^^,^^23^^–^^25^ Each injection consisted of 4 ml of stable isotope-labeled 15  μm microspheres (STERIspheres, BioPal Inc, Medford, Massachusetts) into the left ventricle via a 5 Fr pigtail catheter over 10 s and reference blood (20 ml) was withdrawn at 10  ml/min for analysis (World Precision Instruments, Sarasota, Florida). Brains were excised and sectioned into regions of interest (ROIs), Fig. 1, and analyzed through neutron activation at an independent laboratory (BioPal Inc. Medford, Massachusetts).

### 2.6 MRI Acquisition

All MRI scans were performed on a 3T MRI scanner (Ingenia, Philips) with canines in a headfirst, prone position using a 15 channel receive-only coil. Diffusion weighted images (DWI) for IVIM were collected with 10 *b*-values from 0 to $1000\;s/{\mathrm{mm}}^{2}$ (0, 111, 222, 333, 444, 556, 667, 778, 889, 1000) and three orthogonal directions to ensure a clinically feasible scan time (5:38 min). IVIM scans were prescribed to cover the entire head (two-dimensional single shot EPI, TR/TE = 3056/91 ms, number of slices = 50, slice thickness = 2 mm, FOV = 160 mm/matrix = 128, total scan time = 332 s, SENSE factor = 2, 3 directions). DSC images were acquired using a T1-Bookend method^15^ within 30 min of IVIM (FOV/matrix = 220 mm/224, voxel size = 220/224, single shot, EPI, fat saturated, number of slices = 5, slice thickness = 6 mm, TR/TE = 315/40, flip angle = 75 deg, 200 time points) and delay and dispersion corrected^26^ to return MTT. Diffusion tensor imaging (DTI) for infarct volume progression was prescribed to cover the entire head (TR/TE = 2993/83 ms, FA = 90 deg, BW = 1790 Hz/pixel, no. of slices = 50, slice thickness = 2 mm, FOV = 160 mm/matrix = 128, b-values = 0, $800\;s/{\mathrm{mm}}^{2}$, 32 directions).

### 2.7 Perfusion Comparison

Hemispheric ROIs, shown in Fig. 1, were manually drawn on three central slices on the diffusion image by a trained operator who was blinded to all perfusion values for comparison to microsphere perfusion. Three IVIM slices were averaged to match microsphere thickness (6 mm). To remove high values from misfitting as demonstrated in previous studies,^10^ $D^{*}\geq 0.10$ were excluded from analysis, and the sum squared residuals were analyzed. Manual avoidance of subarachnoid space and ventricles, along with leave-one-out cross validation T2 map thresholds, was used to remove CSF-dominated voxels from analysis. Kwong et al.^13^ suggested using an inversion recovery saturation pulse to remove proposed artifactual high perfusion values in the brain from the glymphatic system. In this study, a simulation was written to explore the effects of this saturation pulse on the blood signal.

### 2.8 Transit Time Comparison

Anatomic middle cortical ROIs post-MCAO were drawn by an operator blinded to physiologic status on pre-processed images of IVIM and DSC ipsilateral and contralateral to the occlusion.

### 2.9 Infarct Volume Comparison

MD analogous to a more widely used apparent diffusion coefficient (ADC) was used to determine the true infarct volume with the automatic threshold of $5.7e\text{-}4^{16}\;{\mathrm{mm}}^{2}/s$. A previously determined automatic IVIM D infarct threshold of $5.15e\text{-}4\;{\mathrm{mm}}^{2}/s$ from leave one out cross validation was applied to calculate IVIM infarct volume.^27^

### 2.10 Statistical Analysis

Linear regression between IVIM and microsphere perfusion was analyzed and pooled across all physiologic states, and the coefficient of determination ($R^{2}$), p-values (p), slopes (m), offsets (b), and corresponding Bland–Altman mean differences and 95% CIs are reported. A mixed effects model was applied with modality as the fixed effect and subject nested as the random effect. Tukey’s boxplots per physiologic state with mean, first and third quartiles, and whiskers were produced with hemispheric paired comparison. Statistical agreement within physiologic states was studied with mean paired difference and Wilcoxon signed rank, and sum squared residuals of the IVIM bi-exponential fits to Eq. (1) are reported. Lin’s concordance correlation coefficient (CCC) was used to determine agreement between multiple quantitative measures incorporating both accuracy and precision between two readings along the 45 deg line through the origin.^28^ In addition, CCCs variability across subjects was shown with the reported subject-wise CCC. All statistical significance was determined at the 5% (p<0.05) level for hypothesis testing.

## 3 Results

**Fig. 2:**
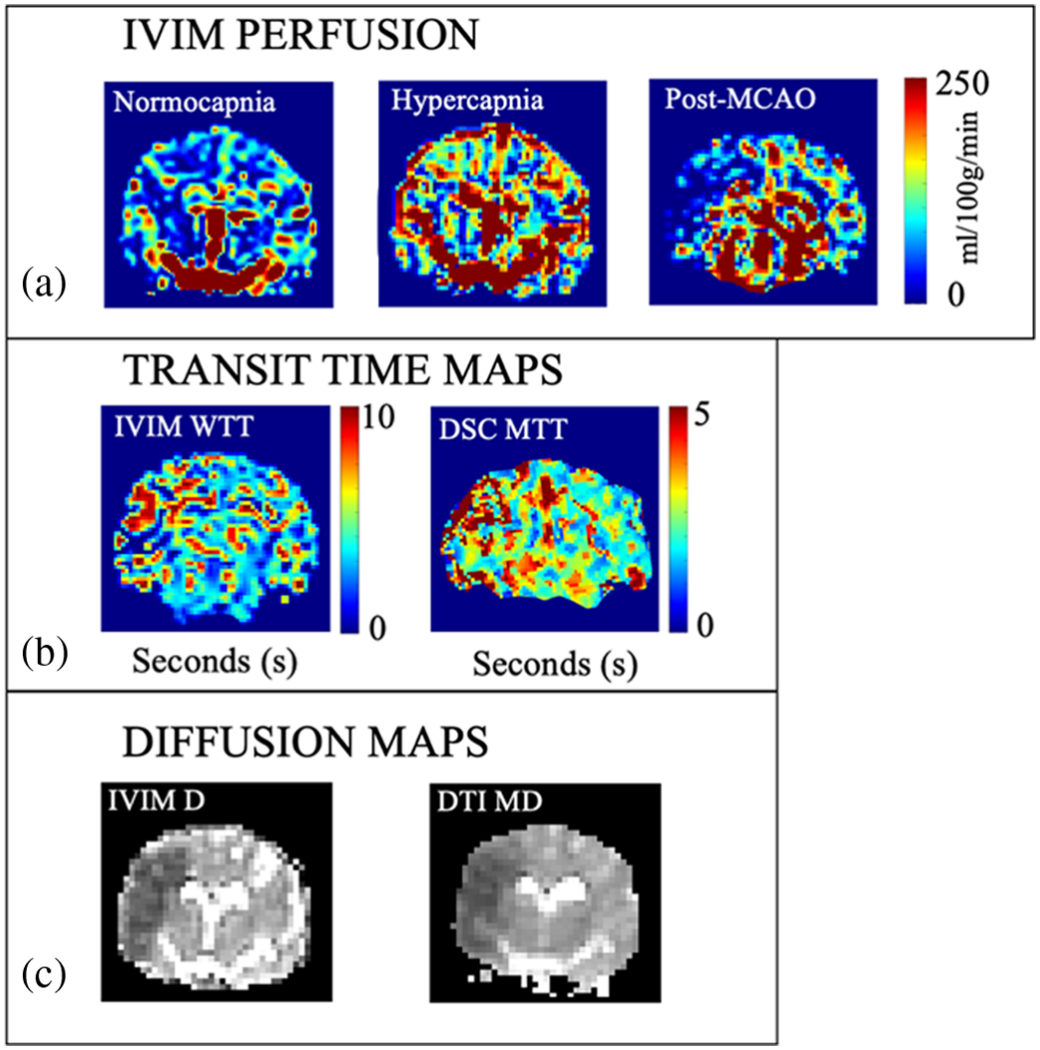
(a) Representative quantitative IVIM perfusion for normocapnia, hypercapnia, and post-MCAO. The qCBF images are shown with a dynamic range of 0 to 250 ml/100 g/min. (b) Transit time maps post-MCAO (left) IVIM WTT and for (right) delay and dispersion corrected DSC MTT. (c) Diffusion images with an IVIM diffusion coefficient image (left) post-MCAO compared with MD map (right) used for the calculation of infarct volume. A case without flow augmentation treatment is provided to highlight the diffusion abnormality post-MCAO.

A total of n=6 (mean age = 0.72y, mean weight = 25.1 kg, 5 female, 1 male) experiments were analyzed with further detail in Table 1. Representative images are shown in Fig. 2(a) for quantitative perfusion images during normocapnia, hypercapnia, and post-MCAO, in Fig. 2(b) for transit time post-MCAO, and in Fig. 2(c) for diffusion-positive maps post-MCAO. Average sum squared residuals of the IVIM signal fits (Table 1) show higher average error at hypercapnia.

**Table 1:** Of the six cases studied, the number of cases available for each physiologic state is shown in the top row, with all measurements within 30 min of each other. Two cases post-MCAO had slices removed from analysis due to zeros being returned by the microsphere vendors. Statistical analysis per physiologic state is shown in the middle row: mean paired difference and corresponding signed rank within each physiologic state, as well as the mean and standard deviation of the sum squared residuals for the voxels included in the analysis ($D^{*}>0.10$) removed following historical threshold. The mixed effects model holding subjects as random variables is shown in the bottom row.

Number of available cases	Normocapnia 5 (case 1 IVIM incomplete)	Hypercapnia 5 (case 4 IVIM incomplete)	Occlusion 5 (case 5 subarachnoid hemorrhage)
**Statistical analysis per physiologic state**	**Mean paired difference** (ml/100 g/min)	**Wilcoxon signed rank** (statistic, p-value)	**Sum squared residuals** (μ±σ)
Normocapnia:	+34.32	6.0, p=0.0005	0.1 ± 0.3
Hypercapnia:	−1.85	79.0, p=0.78	1.0 ± 2.0
Post-MCAO:	+4.02	95.0, p = 0.71	0.002 ± 0.004
**Mixed effects model**	**Mean slope [95% CI]**	**Mean intercept [95% CI]**	
IVIM versus microspheres	0.52 [0.40, 0.64]	54.6 [41.6, 67.7]	

**Fig. 3:**
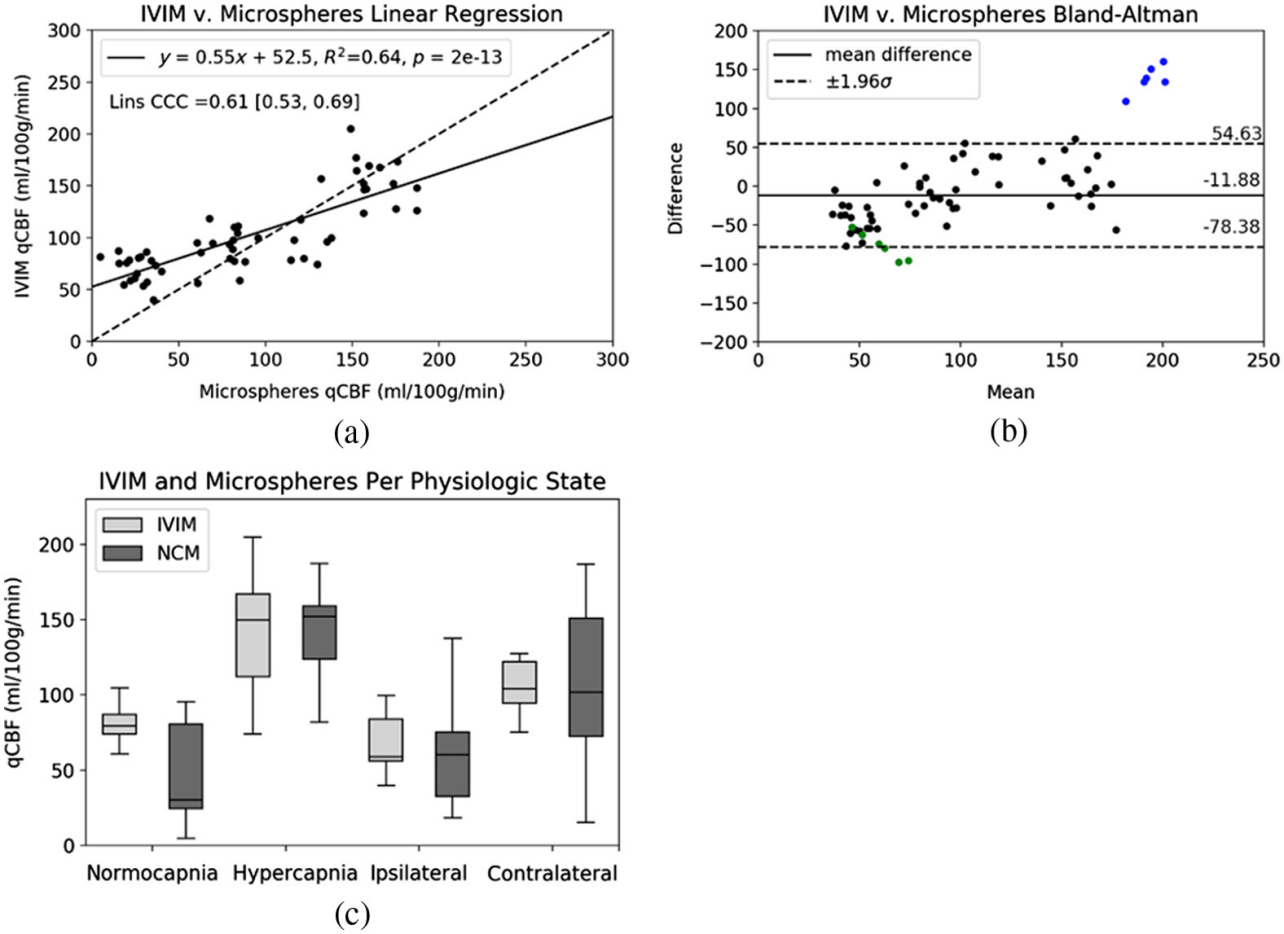
(a) Hemispheric correlation of microsphere qCBF versus quantitative IVIM qCBF across all physiologic states. Solid lines in the linear regression plot represent the line of regression, and a dashed line of unity is shown for reference. (b) Bland–Altman plot with the mean difference as a solid line and dashed lines representing 95% CI. Blue and green data represent the difficulty of dynamic physiology: both were cases with large changes in BP and ${\mathrm{CO}}_{2}$ between IVIM and microsphere injection despite each measurement type being within 30 min of the other. This agrees with the large difference between the two perfusion measurement types in (b). Due to the instability in physiology, these cases were not included in statistical analysis but are shown as outliers in Bland–Altman for the sake of completeness. (c) Tukey’s box-whisker plots of hemispheric values for all physiologic states. Microspheres are called “NCM” for brevity. Normocapnia and hypercapnia include both the left and right hemispheres, whereas occlusion and contralateral are split into corresponding hemispheres. Note that values are considerably higher in the MCAO model due to the use of aggressive flow augmentation.^21^^,^^22^

**Fig. 4:**
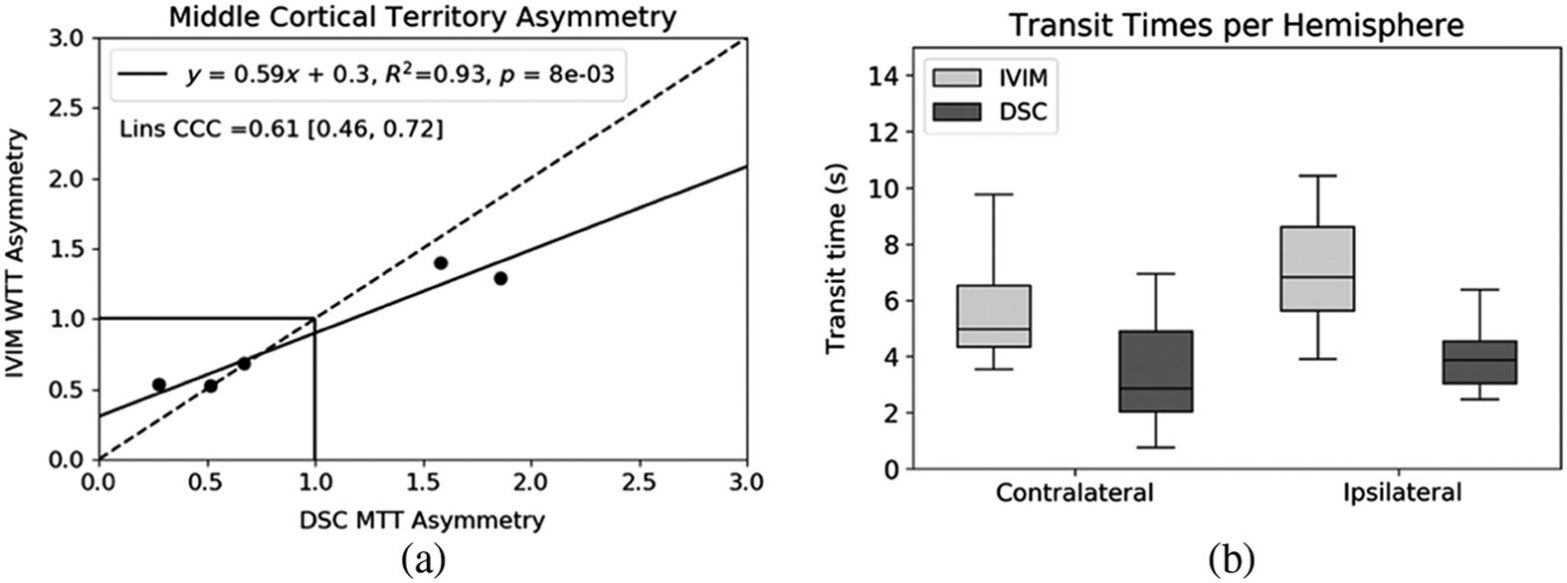
(a) Correlation of middle cortical territory asymmetry (left/right) of the five subjects post-MCAO. (b) Tukey’s box blots of the middle cortical territories post-MCAO showing mean and range of transit times from IVIM and DSC, split into contralateral and ipsilateral hemispheres.

Figures 3(a) and 3(b) show linear regression, Lin’s CCC, and corresponding Bland–Altman of IVIM and microsphere perfusion. Tukey’s box plots in Fig. 3(c) demonstrate average and range of perfusion at normocapnia, hypercapnia, and post-MCAO split into ipsilateral and contralateral hemispheres. There were no significant differences in hypercapnia or post-occlusion hemispheres, but a statistically significant difference was seen at normocapnia (Table 1). The mixed effects model showed correlation existed holding subjects as random effects as well, supporting IVIM sensitivity to changing physiology (Table 1). Linear regression of middle cortical asymmetry of IVIM WTT post-occlusion returned correlation to DSC MTT [Fig. 4(a)] and Tukey’s Box plots show an expected increase in transit time on the ipsilateral hemispheres, though with a different mean and range of values [Fig. 4(b)]. Automatic infarct volume from IVIM returned strong linear correlation to MD (slope=1.01, intercept=0.58, $R^{2}=0.71$, CCC=0.79). Subject-wise Lin’s CCC for cases with all slices analyzed ranged from 0.26 to 0.86, with a mean of 0.56. The simulation of inversion recovery spin echo (TR = 5000 ms, TE = 37 ms) for CSF suppression of the remaining transverse signal from CSF and blood returned inversion time of 1850 ms to be the best time for CSF suppression. However, this optimal TI for CSF suppression also suppressed the blood signal to 18% of the original when both T1 and T2 are included.

In the capillary geometry based analysis, we found linear regression (slope = 0.60, intercept = 57.2, $R^{2}=0.64$, Lin’s CCC = 0.68 [0.59, 0.75]) with a Wilcoxon signed-rank statistic across all states equal to 308 and p=0.0006. BA analysis found a mean difference and 95% CI of −20[−79,37] compared to the water transit time model, which found mean difference and 95% CI of −11[−78, 54]. The WTT model demonstrates comparable sensitivity and an improved mean difference without the assumptions of capillary dimension in vessel-dependent models.

## 4 Discussion

This study found that a 5-min IVIM 10 b-value sequence can produce quantitative cerebral perfusion values via WTT in a clinically acceptable scan time across a range of relevant physiologic conditions. The sensitivity of IVIM perfusion metrics to physiologic changes in tissue suggest that IVIM images can have impact in the study and progression tracking of cerebrovascular disease.^9^ Furthermore, IVIM images are sensitive to changes in qCBF in response to ${\mathrm{CO}}_{2}$ inhalation (cerebrovascular reactivity), without repeated administration of the contrast agent, an advantage in the evaluation of chronic disease.^10^^,^^14^ Further, the study suggests a possible quantification factor that relies solely on assumed randomly oriented isotropic diffusing water transport and not on organ-dependent capillary segment length parameters. All comparisons between IVIM and microspheres across three physiologic states returned strong linear regression correlation and were further supported by the agreement shown with Lin’s CCC analysis.

Prior studies compare IVIM cerebral perfusion to MR CBF measurements with varying levels of success.^10^^,^^29^^,^^30^ Several studies compare DSC CBV and IVIM f,^10^^,^^29^ and one study ($R^{2}=0.42$) compares relative IVIM CBF fD* and relative DSC CBF.^30^ In comparison, this study found a strong correlation ($R^{2}=0.64$) of quantitative IVIM CBF fD* in ml/100 g/min to neutron capture microsphere qCBF in ml/100 g/min. Mixed effects modeling showed that this sensitivity exists holding subjects as random effects as well (Table 1). This linear regression suggests sensitivity across physiologic states, and paired agreement within physiologic states supports the quantification of hemispheric perfusion without capillary parameter assumptions in a multi-day controlled pre-clinical model, which to the best of our knowledge has not been published previously. By showing correlation to an absolute quantitative method, the work supports a method that allows absolute comparison across multiple subjects, days, and experimental groups. In comparison, standard relative perfusion requires comparison to an internal reference that is confounded if the internal reference varies.^22^

However, the correlation also highlights an example of the remaining difficulty of CSF removal: partial volume contamination of CSF may falsely add to the perfusion signal at normocapnia, shown in Fig. 3(c), and lead to higher average perfusion with p<0.05 at normocapnia, though this study does not explore this. Although the standard IVIM model is bi-exponential, assuming b-values from 222 to 1000 is mono-exponential, and b-values <222 are mono-exponential, the examination of tri-exponential behavior with more than just two fluid compartments is needed. Notably, although not explored in this paper, voxels with partial volume contamination such as those along the periphery of the cortex and close to the boundaries of the ventricles showed tri-exponential behavior, potentially from a three-compartment model. Further, the higher residuals at hypercapnia show increased perfusion causing a poorer bi-exponential fit, a potential factor in the lower sensitivity of IVIM to increased perfusion.

WTT used to quantify perfusion showed linear correlation with delay and dispersion corrected DSC MTT asymmetry in the affected middle cortical territory post-MCA occlusion. In the interpretation of the DSC or CTP perfusion values clinically, time-based metrics used in bolus track perfusion calculation are often used due to the high sensitivity that these measures have to pathology. As IVIM and bolus-track DSC are very different approaches, the “water transit time” is derived to be an analog of MTT to study if IVIM based on fast water diffusion relates to MTT as determined by blood flow through a vascular bed as determined by DSC. The correlation of WTT and MTT provides another quantifiable metric to link the two methods for quantifying perfusion. Ii is important to note that comparison of IVIM WTT to DSC requires that DSC undergoes delay and dispersion correction, i.e., a local AIF, to account for IVIM AIF-independence. Without this correction of DSC, the correlation of qCBF and transit time would be weaker. The difference in transit time range observed is notable [Fig. 3(b)], likely due to the difference in properties being measured and endogenous versus exogenous measurement. Further, zeros are noted where $D^{*}$ is small as its inverse (WTT) is inversely proportionally high. To prevent influencing the results, these voxels are assigned 0 in Fig. 2(b), rather than being assigned an estimated WTT, and are not included in the analysis. The agreement of transit time supports the IVIM model with the assumption of instantaneous bolus mimicking the central volume principle through isotropic Gaussian diffusion if the DSC perfusion uses a local AIF.

Previous studies have also reported positive correlation between ADC and IVIM D in ischemic and normal regions ($R^{2}=1$, $R^{2}=1$)^31^ and ($R^{2}=0.98$, $R^{2}=0.81$).^32^ Although this study’s agreement was lower than prior studies (slope = 1.01, $R^{2}=0.79$), it should be noted that the IVIM and DTI scans that were compared were taken from separate MR scans during a period of dynamic infarct expansion, whereas the previous studies used the same images to get ADC and IVIM D. The use of two separate scans and sequences for IVIM D and MD infarct volume was chosen to best compare IVIM D and a separate standard clinical infarct volume. This combined with the IVIM qCBF from the same IVIM scan, not included in previous studies, demonstrates that the simultaneous perfusion and diffusion components from a single IVIM bi-exponential fit agreed with corresponding perfusion and diffusion reference standard values.

IVIM imaging exploits the fact that diffusion-weighting is sensitive to water motion and consequently is able to identify where in the brain water is sequestered inside cells by cytotoxic edema.^33^ At very low b-values ($b\leq 200\;s/{\mathrm{mm}}^{2}$), diffusion-weighting is predominantly sensitive to faster water motion, such as capillary level blood flow.^11^^,^^14^^,^^34^^–^^36^ Questions remain regarding the origin of fast water motion signal, with Kwong et al.^13^ showing that the use of an inversion recovery pulse significantly reduced the cortical gray matter pseudo-diffusion fraction, which suggests that much of the bi-exponential behavior resulted from CSF contamination rather than perfusion.^13^ The simulation of inversion recovery spin echo suppression shows that it will suppress both CSF and blood from both T1 and T2 effects. Further, following the Monro–Kellie hypothesis, at hypercapnia, the dilation of cerebral capillaries prompts an increase in cerebral blood volume and CBF and a decrease in CSF to maintain intracranial pressure.^37^ Therefore, if signal in the fast-water compartment of the bi-exponential IVIM signal were due solely to CSF contamination, hypercapnia from ${\mathrm{CO}}_{2}$ inhalation should return a decrease in the fast-water compartment, which was not observed in this study [Fig. 3(c)]. Avoidance of voxels in the ventricles and subarachnoid space from diffusion maps, along with leave-one-out cross validation T2 map thresholds, reduced the effect of human bias in ROI selection. This study provides evidence that observed IVIM signal changes reflect cytotoxic edema and tissue perfusion. However, endogenous contrast prevents proof of signal origin, and partial volume effects require further study especially in disease states.

Arterial spin labeling (ASL) is a well-established, widely available, and fully quantitative means of imaging cerebral perfusion noninvasively.^38^ However, with ASL, delayed arterial arrival times due to proximal vessel occlusion in stroke studies remains a challenge.^39^^,^^40^ As such, IVIM being independent of arterial input functions or bolus kinetics is a singificant benefit of the sequence in comparison with both ASL and DSC-MRI when measuring neurovascular disease. Futher, IVIM is able to provide simultaneous identification of cytotoxic edema, unlike ASL or DSC-MRI.

This study is not without limitations. The highly dynamic physiology created challenges for the acquisition of normocapnia, hypercapnia, and post-MCAO with MRI and invasive neutron capture microspheres within tight timing windows. Two cases showing large disagreement between IVIM and microsphere perfusion also display differences in BP and ${\mathrm{CO}}_{2}$ between the two measurements that agree with the discrepancy in perfusion agreement that is shown [Fig. 3(b)], though the causality cannot be proven. This supports the physiologic change between MRI and microspheres that contribute to scatter in the linear regression. Difficulty in perfect alignment of physical brain cutting segmentation and discrete MR imaging also contributes to added noise in the linear regression. Although sensitivity to physiologic change is observed, IVIM sensitivity appears weaker than the perfusion effects observed with microspheres. Notably, normocapnia returns higher IVIM perfusion compared with microspheres, possibly due to partial volume contamination from CSF. The exponential fitting error, especially regarding pseudo-diffusion coefficient, is a continuing problem. Although limits were placed to maintain pseudo-diffusion coefficients in the expected range following the historical literature,^30^ pseudo-diffusion error will have large effects due to the inverse relation to WTT, and the error is observed to increase at hypercapnia. Further, some data were lost beyond our control due to vendor error and dynamic physiology, highlighting the complexity of microsphere perfusion. Finally, translatability of this perfusion to humans is not known.

## 5 Conclusions

Diffusion positive volumes and quantitative cerebral perfusion from IVIM agreed with reference standard values over a range of physiologic conditions. The sensitivity of IVIM provides evidence that observed signal changes reflect cytotoxic edema and tissue perfusion. IVIM images were acquired from 5 min of non-contrast DWI and quantified with WTT independent of capillary geometric assumptions and returned agreement to neutron capture microspheres across a wide range of physiologic states, such as normocapnia, hypercapnia, and infarction. This supports the further development and refinement of IVIM for measuring noninvasive quantitative perfusion and diffusion.

## 6 Appendix

Step-by-step derivation of WTT, the time at which 50% of the original molecules have diffused with diffusion-coefficient $D^{*}$ beyond a unit boundary, is given. In other words, this is an equation for the concentration of a species to calculate the time taken for the initial concentration to be reduced by half. If particles begin as a delta function in the center of a voxel, the way tracer kinetics is modeled as an instantaneous bolus in DSC, following the law of diffusion the distribution of particles will change over time according to a normal distribution as a function of $\sigma =\sqrt{2Dt}$, where D is the diffusion coefficient and t is the time it has been diffusing. WTT can be solved by integrating over one unit of a three-dimensional Gaussian centered at zero and setting it equal to 0.50 as follows:

$$\int_{0}^{2\pi }\int_{0}^{\pi }\int_{0}^{.5}\frac{1}{\sqrt{{\left(2\pi \right)}^{3}{\left(2D^{*}\mathrm{WTT}\right)}^{3}}}e^{*-\frac{r^{2}}{4D\mathrm{WTT}}}r^{2}\;\sin\;\theta dr\;d\theta \;d\phi =0.50\;\frac{4\pi }{{\left(\sqrt{4\pi D^{*}\mathrm{WTT}}\right)}^{3}}\int_{0}^{.5}e^{-\frac{r^{2}}{4D^{*}\mathrm{WTT}}}r^{2}dr=0.50.$$ (4)

The concentration over time will decrease as water diffuses out of the sphere with diffusion coefficient $D^{*}[\frac{{\mathrm{mm}}^{2}}{s}]$. Solving for WTT is shown below. For simplicity, s is substituted for $4D^{*}\mathrm{WTT}$, and the integral is solved by integration by parts. The limits of integration are set from 0 to 0.5 mm because the instantaneous impulse function is set at the center of a unit volume sphere and returns the following:

$$\int_{0}^{.5}e^{-\frac{r^{2}}{s}}r^{2}dr=-r{\left(\frac{s}{2}e^{-\frac{r^{2}}{s}})\right|}_{0}^{.5}+\int_{0}^{.5}\frac{s}{2}e^{-\frac{r^{2}}{s}}dr=-.25s(e^{-\frac{.25}{s}})+\frac{\sqrt{\pi }}{4}s^{\frac{3}{2}}\;\mathrm{erf}(\frac{.5}{\sqrt{s}}).$$

Plugging this into Eq. (4) returns:

$$\begin{array}{c} \frac{4\pi }{(\pi s{)}^{\frac{3}{2}}}[-.25s(e^{-\frac{.25}{s}})+\frac{\sqrt{\pi }}{4}s^{\frac{3}{2}}\;\mathrm{erf}(\frac{.5}{\sqrt{s}})]=0.5\\ -\frac{1}{\sqrt{\pi s}}e^{-\frac{.25}{s}}+\mathrm{erf}(\frac{.5}{\sqrt{s}})=0.5. \end{array}$$

**Fig. 5:**
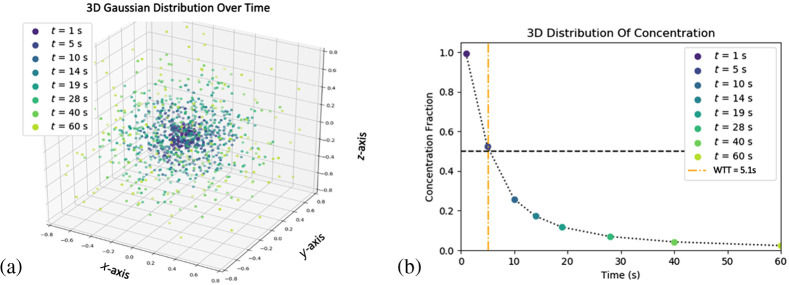
(a) A simulation of 3D diffusion for a voxel with a pseudo-diffusion coefficient of $D^{*}=0.009$ with the distribution plotted as it spreads over time. (b) The corresponding concentration as it drops over time, with the orange line showing the WTT at which the concentration is 50% of the original. This is an example of the transit time that this study uses to quantify IVIM perfusion using the central volume principle.

Due to the nature of erf(x), this is not analytically solvable. Therefore, the use of a numerical approximation of erf(x) is required. The approximation of erf(x) from equation (7.1.27) of Ref. 41 returns an error $\leq 5e^{-4}$ and is given as follows (Fig. 5):

$$\mathrm{erf}(x)=\frac{2}{\sqrt{\pi }}\int_{0}^{x}e^{-t^{2}}dt=1-{\left(1+0.278393x+0.230389x^{2}+0.000972x^{3}+0.078108x^{4}\right)}^{-4}.$$

Plugging this in returns the following equation, which can be solved for s:

$$-\frac{1}{\sqrt{\pi s}}e^{-\frac{0.25}{s}}+[1-(1+0.278393(\frac{0.5}{\sqrt{s}})+0.230389{\left(\frac{0.5}{\sqrt{s}}\right)}^{2}\;{+0.000972{\left(\frac{0.5}{\sqrt{s}}\right)}^{3}+0.078108{\left(\frac{0.5}{\sqrt{s}}\right)}^{4})}^{-4}]=0.5\approx 0.21.$$

This can be checked by plugging $4D^{*}\mathrm{WTT}=s\approx 0.21$ into Eq. (4) as follows:

$$\frac{4\pi }{{\left(\sqrt{0.21\pi }\right)}^{3}}\int_{0}^{0.5}e^{-\frac{r^{2}}{0.21}}r^{2}dr=0.50281.$$

Returning to Gaussian diffusion parameters, if we substitute $\sigma =\sqrt{2D^{*}\mathrm{WTT}}=\frac{\sqrt{s}}{\sqrt{2}}$, we can solve for $\sigma =\frac{\sqrt{0.21}}{\sqrt{2}}$ and get σ≈0.32.

Solving this for WTT in terms of D returns

$$\mathrm{WTT}=\frac{{\left(0.32\right)}^{2}}{2D^{*}}.$$

This is the same as if we solved for WTT in terms of $D^{*}$ straight from the approximation of s, but in terms of σ of the the 3D Gaussian. With this WTT derived and explained intuitively, we can plug this into the central volume principle (blood flow = blood volume/transit time) to return the calibration coefficient for the fast-moving component of IVIM volume f and pseudo-diffusion coefficient $D^{*}$:

$$\mathrm{qCBF}=\frac{\mathrm{CBV}}{\mathrm{WTT}}=\frac{f\times f_{w}}{\rho }\frac{2D^{*}}{(0.32{)}^{2}}.$$

It should be emphasized that this is an approximation that is valid when capillary perfusion direction is randomly oriented and isotropic and dominates the anisotropic signal. The water content fraction of a voxel $f_{w}$, the density ρ, the numerical estimation used for solving the erf(x), and the assumption of it being a sphere all contribute to the quantification factor above. This is the trade-off in assumptions compared with capillary bed network assumptions, such as capillary segment length, diameter, and path length, that are organ-dependent and vary under physiologic stress. Using the WTT based perfusion model to quantify IVIM perfusion returns the following:

$${\mathrm{qCBF}}_{\mathrm{WTT}}=fD^{*}\times \frac{2f_{w}}{\rho {\left(0.32\right)}^{2}}\;=fD^{*}[{\mathrm{mm}}^{2}/s]\times \frac{2(0.79)}{1.0[\frac{g}{\mathrm{mL}}]{\left(0.32[\mathrm{mm}]\right)}^{2}}\times 100\times 60\frac{s}{\min}\;\approx fD^{*}\times 93,000[\frac{\mathrm{ml}}{100}g/\min]$$

## Data Availability

The post processing code was written in MATLAB and is stored in Github and readily available. All images are stored local in easily read DICOM format. Physiologic monitoring and drug administration are recorded in Microsoft Excel worksheets. These records are time-stamped to allow for cross referencing with image acquisition times (provided in the DICOM headers). The archive of all data are available by request from the corresponding author.
